# Supplementary material for: Theory and Applications of the (Cardio) Genomic Fabric Approach to Post-Ischemic and Hypoxia-Induced Heart Failure
Source: J Pers Med. 2022 Jul 29;12(8):1246. doi: 10.3390/jpm12081246 (PMC9410512; doi:10.3390/jpm12081246)
Supplement: Supplementary file 1 [file jpm-12-01246-s001.zip › jpm-1794743-supplementary.pdf]

Table S1: Average expression (AVE) normalized to the median gene expression levels of 34 inflammatory response genes, *Ank2* and the genes with the largest expression level in the entire transcriptome after 2 and 4 weeks exposure to normal atmospheric conditions (N2, N4), chronic intermittent hypoxia (I1, I2) and chronic constant hypoxia (C2, C4). Light gray background indicates the genes with the largest expression level within the selection while the darker gray indicates the gene with the largest expression level in each condition.

Symbol	Gene	N2	I2	C2	N4	I4	C4
Atrn	Attractin	0.27	0.30	0.24	0.31	0.21	0.36
Ccl22	Chemokine (C-C motif) ligand 22	0.29	0.29	0.27	0.28	0.25	0.26
Cx3cl1	Chemokine (C-X3-C motif) ligand 1	0.16	0.15	0.29	0.21	0.24	0.23
Cxcl4	Chemokine (C-X-C motif) ligand 4	0.27	0.12	0.41	0.23	0.33	0.28
Ifnar1	Interferon (alpha and beta) receptor 1	0.27	0.20	0.58	0.24	0.29	0.27
Ifngr2	Interferon gamma receptor 2	1.23	1.07	1.21	1.05	0.94	0.76
zIl10ra	Interleukin 10 receptor, alpha	0.18	0.14	0.15	0.11	0.13	0.08
Il11ra1	Interleukin 11 receptor, alpha chain 1	3.47	4.94	4.48	6.86	5.94	5.52
Il16	Interleukin 16	0.20	0.17	0.27	0.14	0.12	0.21
Il17b	Interleukin 17B	0.50	0.38	0.10	0.49	0.42	0.29
Il1f6	Interleukin 1 family, member 6	5.44	4.18	5.42	7.21	7.19	6.80
Il28ra	Interleukin 28 receptor alpha	0.29	0.16	0.18	0.25	0.16	0.15
Il31ra	Interleukin 31 receptor A	0.88	0.47	1.78	0.89	0.85	1.32
Il4	Interleukin 4	0.71	0.49	0.30	0.26	0.27	0.25
Il6st	Interleukin 6 signal transducer	0.36	0.42	0.34	0.32	0.41	0.51
Il7r	Interleukin 7 receptor	2.14	3.01	1.75	3.04	3.27	2.85
Lif	Leukemia inhibitory factor	0.49	0.48	0.61	0.35	0.45	0.33
Ly86	Lymphocyte antigen 86	0.21	0.19	0.11	0.16	0.16	0.15
Mif	Macrophage migration inhibitory factor	0.85	1.35	2.00	0.82	0.62	0.73
Nfkbiz	Nuclear factor of kappa light polypeptide gene enhancer in B-cells inhibitor, zeta	1.14	1.29	0.85	1.45	1.66	1.20
Prlpk	Prolactin-like protein K	2.06	1.83	0.88	2.78	3.31	2.45
Prlr	Prolactin receptor	10.23	11.07	5.99	7.25	7.12	4.05
Ptpn6	Protein tyrosine phosphatase, non-receptor type 6	0.32	0.13	0.48	0.26	0.20	0.36
Reg3g	Regenerating islet-derived 3 gamma	0.34	0.33	0.13	0.40	0.43	0.16
Repin1	Replication initiator 1	0.96	0.29	0.70	0.55	0.59	0.51
Rqcd1	Rcd1 (required for cell differentiation) homolog 1 (S. pombe)	0.22	0.15	0.30	0.23	0.19	0.33
Scgb3a1	Secretoglobin, family 3A, member 1	1.90	1.50	1.36	4.03	2.55	4.77
Scye1	Small inducible cytokine subfamily E, member 1	0.24	0.38	0.43	0.18	0.15	0.09
Stab1	Stabilin 1	0.26	0.33	0.21	0.20	0.20	0.10
Tlr4	Toll-like receptor 4	0.14	0.18	0.25	0.18	0.19	0.08
Tlr7	Toll-like receptor 7	3.02	2.71	4.08	4.82	3.49	4.61

Tollip	Toll interacting protein	0.24	0.19	0.34	0.17	0.21	0.15
Ttll12	Tubulin tyrosine ligase-like family, member 12	2.96	4.69	3.88	4.57	3.37	4.05
Xcl1	Chemokine (C motif) ligand 1	0.30	0.26	0.23	0.33	0.29	0.40
Ank2	Ankyrin 2, brain	0.26	0.20	0.32	0.22	0.36	0.34
Hspb6	Heat shock protein, alpha-crystallin-related, B6	14.52	16.51	14.12	23.04	14.11	20.58
Nr1i3	Nuclear receptor subfamily 1, group I, member 3	19.61	11.60	5.42	24.96	18.97	21.06

Table S2: Relative expression variation (REV) of 34 inflammatory response genes, *Ank2* and the most stably expressed genes in the entire transcriptome after 2 and 4 weeks exposure to normal atmospheric conditions (N2, N4), chronic intermittent hypoxia (I1, I2) and chronic constant hypoxia (C2, C4). Light gray background indicates the genes with the largest expression control (i.e. lowest REV) level within the selection while the darker gray indicates the gene with the largest control in the entire condition. Bold Italics indicate the largest variabilities in the selected gene subset.

Symbol	Gene	N2	I2	C2	N4	I4	C4
Atrn	Attractin	5.6	20.9	13.9	43.1	12.9	42.3
Ccl22	Chemokine (C-C motif) ligand 22	13.0	15.2	31.0	5.2	23.0	27.2
Cx3cl1	Chemokine (C-X3-C motif) ligand 1	29.9	28.7	13.8	30.8	20.0	14.5
Cxcl4	Chemokine (C-X-C motif) ligand 4	30.6	19.5	14.2	33.5	31.9	36.6
Ifnar1	Interferon (alpha and beta) receptor 1	14.7	45.6	16.3	58.7	20.2	16.0
Ifngr2	Interferon gamma receptor 2	14.1	29.3	16.0	40.0	18.2	36.8
Il10ra	Interleukin 10 receptor, alpha	21.9	66.2	27.6	34.8	21.4	47.3
Il11ra1	Interleukin 11 receptor, alpha chain 1	18.2	46.2	19.5	49.5	10.3	28.0
Il16	Interleukin 16	13.1	9.1	8.7	16.9	22.8	54.0
Il17b	Interleukin 17B	15.3	30.3	35.5	12.0	13.6	24.2
Il1f6	Interleukin 1 family, member 6	16.9	22.9	38.5	26.1	15.5	34.0
Il28ra	Interleukin 28 receptor alpha	22.6	15.5	36.1	10.7	33.8	32.0
Il31ra	Interleukin 31 receptor A	33.2	62.3	22.1	3.2	22.7	34.1
Il4	Interleukin 4	48.5	15.3	47.4	22.1	18.7	39.4
Il6st	Interleukin 6 signal transducer	8.8	32.8	19.8	14.6	27.0	33.8
Il7r	Interleukin 7 receptor	12.2	68.2	31.2	75.7	18.1	28.4
Lif	Leukemia inhibitory factor	4.0	4.8	20.5	13.3	14.9	19.1
Ly86	Lymphocyte antigen 86	15.7	30.1	29.6	32.2	19.0	50.0
Mif	Macrophage migration inhibitory factor	7.4	39.4	14.1	51.2	41.6	41.9
Nfkbiz	Nuclear factor of kappa light polypeptide gene enhancer in B-cells inhibitor, zeta	10.4	12.3	18.9	12.4	16.3	36.5
Prlpk	Prolactin-like protein K	7.9	21.9	16.2	35.4	32.7	25.3
Prlr	Prolactin receptor	15.8	12.1	6.3	29.9	51.8	52.7
Ptpn6	Protein tyrosine phosphatase, non-receptor type 6	38.0	40.0	25.3	27.8	34.6	35.8
Reg3g	Regenerating islet-derived 3 gamma	19.6	38.5	46.0	38.4	42.7	10.6
Repin1	Replication initiator 1	27.8	29.6	24.0	7.7	38.8	37.8
Rqcd1	Rcd1 (required for cell differentiation) homolog 1 (S. pombe)	22.0	23.4	30.4	24.4	19.9	42.0
Scgb3a1	Secretoglobin, family 3A, member 1	35.3	22.8	16.6	10.5	45.3	8.9
Scye1	Small inducible cytokine subfamily E, member 1	5.6	44.1	30.8	64.7	36.7	41.0

Stab1	Stabilin 1	26.3	20.1	9.5	29.2	18.8	39.6
Tlr4	Toll-like receptor 4	20.1	32.3	14.6	40.5	27.2	8.0
Tlr7	Toll-like receptor 7	10.5	18.7	22.3	16.9	11.8	5.0
Tollip	Toll interacting protein	8.9	45.5	24.6	56.9	15.3	22.4
Ttll12	Tubulin tyrosine ligase-like family, member 12	15.0	25.5	32.3	30.8	8.9	46.2
Xcl1	Chemokine (C motif) ligand 1	15.2	10.6	3.4	17.1	18.8	16.3
Ank2	Ankyrin 2, brain	31.5	52.0	17.7	64.6	23.3	21.8
Ankrd15	Ankyrin repeat domain 15	0.5	53.3	18.9	47.0	6.2	16.0
Tubg1	Tubulin, gamma 1	4.7	0.4	10.7	19.4	62.5	19.1
Dmkn	Dermokine	16.3	11.6	1.1	11.9	14.3	57.3
Mrpl15	Mitochondrial ribosomal protein L15	12.0	11.8	14.5	1.3	15.3	16.8
Qtrtd1	Queuine tRNA-ribosyltransferase domain containing 1	11.9	14.2	9.7	25.2	1.3	32.1
Arid2	AT rich interactive domain 2 (Arid-rfx like)	20.6	37.4	8.9	37.9	12.0	0.6

Table S3: Expression coordination of 34 inflammatory response genes with *Ank2* after 2 and 4 weeks exposure to normal atmospheric conditions (N2, N4), chronic intermittent hypoxia (I1, I2) and chronic constant hypoxia (C2, C4). Red/blue/yellow background indicates statistically ($p < 0.05$) significant synergistic/antagonistic/independent expression of that gene with *Ank2*, while black background indicates perfect positive correlation (COR =1) of *Ank2* with itself.

Symbol	Gene	N2	I2	C2	N4	I4	C4
Atrn	Attractin	-0.567	0.409	-0.944	0.674	0.839	0.160
Ccl22	Chemokine (C-C motif) ligand 22	-0.677	0.837	-0.349	0.001	-0.483	0.620
Cx3cl1	Chemokine (C-X3-C motif) ligand 1	0.937	0.900	0.355	0.962	0.793	-0.541
Cxcl4	Chemokine (C-X-C motif) ligand 4	0.981	-0.416	-0.025	0.909	-0.227	0.783
Ifnar1	Interferon (alpha and beta) receptor 1	0.768	0.920	0.897	0.991	0.776	0.844
Ifngr2	Interferon gamma receptor 2	0.929	0.932	0.725	0.950	0.295	0.959
Il10ra	Interleukin 10 receptor, alpha	-0.412	-0.828	-0.616	0.529	-0.418	-0.482
Il11ra1	Interleukin 11 receptor, alpha chain 1	0.951	-0.979	-0.885	-0.998	0.212	-0.809
Il16	Interleukin 16	-0.920	0.300	0.508	0.484	0.531	0.809
Il17b	Interleukin 17B	0.581	-0.964	0.092	0.144	-0.472	-0.190
Il1f6	Interleukin 1 family, member 6	-0.990	0.779	-0.211	-0.993	-0.598	0.010
Il28ra	Interleukin 28 receptor alpha	0.920	-0.866	-0.074	0.802	-0.119	-0.646
Il31ra	Interleukin 31 receptor A	0.989	0.972	0.765	0.501	0.635	0.679
Il4	Interleukin 4	-0.910	0.265	0.092	0.009	0.753	-0.037
Il6st	Interleukin 6 signal transducer	0.858	0.903	0.994	0.681	0.574	-0.762
Il7r	Interleukin 7 receptor	-0.925	-0.975	-0.910	-0.969	-0.598	-0.047
Lif	Leukemia inhibitory factor	0.764	0.701	0.179	-0.649	0.761	0.118
Ly86	Lymphocyte antigen 86	-0.996	-0.972	-0.117	0.814	0.609	0.402
Mif	Macrophage migration inhibitory factor	-0.369	-0.885	0.682	0.873	-0.544	0.470
Nfkbiz	Nuclear factor of kappa light polypeptide gene enhancer in B-cells inhibitor, zeta	-0.891	-0.832	-0.413	-0.730	-0.042	-0.946

Prlpk	Prolactin-like protein K	-0.313	-0.780	-0.955	0.707	0.059	-0.781
Prlr	Prolactin receptor	-0.988	-0.543	-0.550	0.370	-0.127	-0.990
Ptpn6	Protein tyrosine phosphatase, non-receptor type 6	-0.940	-0.679	-0.519	0.530	-0.323	0.111
Reg3g	Regenerating islet-derived 3 gamma	0.923	-0.943	-0.017	-0.975	-0.239	-0.296
Repin1	Replication initiator 1	-0.942	0.683	-0.856	-0.676	-0.357	0.688
Rqcd1	Rcd1 (required for cell differentiation) homolog 1 (S. pombe)	0.820	0.828	-0.217	0.814	0.034	0.687
Scgb3a1	Secretoglobin, family 3A, member 1	-0.944	-0.774	-0.951	-0.471	-0.108	-0.287
Scye1	Small inducible cytokine subfamily E, member 1	0.213	0.684	0.776	0.920	-0.580	-0.052
Stab1	Stabilin 1	-0.724	-0.639	0.341	0.913	-0.860	-0.229
Tlr4	Toll-like receptor 4	0.789	0.948	-0.831	-0.980	-0.172	-0.595
Tlr7	Toll-like receptor 7	-0.927	0.607	-0.476	-0.022	-0.446	0.304
Tollip	Toll interacting protein	-0.026	0.997	0.931	0.983	0.950	0.980
Ttll12	Tubulin tyrosine ligase-like family, member 12	-0.887	-0.919	-0.183	0.524	-0.183	-0.638
Xcl1	Chemokine (C motif) ligand 1	0.757	-0.852	-0.825	0.184	0.342	0.252
Ank2	Ankyrin 2, brain	1.000	1.000	1.000	1.000	1.000	1.000
